# Memory improvement in senile rats after prebiotic and probiotic supplementation is not induced by GLP‐1

**DOI:** 10.1111/cns.13951

**Published:** 2022-09-02

**Authors:** Gabriela Andrea Servín‐Casas, Alejandra Romo‐Araiza, Gabriela Gutierrez‐Salmean, Enrique Martinez‐Solis, Andrea Paola Ibarra‐García, Yolanda Cruz‐Martinez, Roxana Rodriguez‐Barrera, Elisa García, Diego Incontri‐Abraham, Antonio Ibarra

**Affiliations:** ^1^ Centro de Investigación en Ciencias de la Salud (CICSA), FCS Universidad Anáhuac México Campus Norte Huixquilucan, Edo. de México Mexico

**Keywords:** microbiota‐gut‐brain axis, prebiotics, probiotics, spatial memory, symbiotic

## Abstract

**Introduction:**

The mechanism underlying the memory improvement induced by prebiotic and probiotic supplementation remains unclear. Glucagon‐like peptide type 1 (GLP‐1) could play an important role since it is induced by prebiotics and enhances memory and learning.

**Aims:**

We correlated the levels of GLP‐1 with spatial memory in senile animals to determine its role in memory improvement after prebiotic and probiotic supplementation.

**Methods:**

Senile rats were randomly assigned to four groups: (1) water (control); (2) *Enterococcus faecium* (probiotic); (3) agave inulin (prebiotic); and (4) *E. faecium* + agave inulin (symbiotic). Each supplement was administered by an orogastric cannula for 5 weeks. In the fifth week, spatial memory was assessed using the Morris Water Maze test (MWM). We extracted the hippocampus, intestine, and serum. GLP‐1 levels were quantified by enzyme‐linked immunosorbent assay.

**Results:**

A significant decrease in escape latency time in the MWM was observed in all groups treated with supplements. The symbiotic group achieved the highest reduction (15.13 s ± 6.40) (*p* < 0.01). We did not find a significant increase in GLP‐1 levels nor a direct correlation of its levels with spatial memory improvement (*p* > 0.05).

**Conclusion:**

Prebiotic and probiotic supplementation improved spatial memory in senile animals. However, this beneficial effect did not correlate with GLP‐1 levels.

## Graphical abstract

GLP‐1 levels do not correlate with spatial memory in symbiotic‐treated rats. No significant correlation was observed between spatial memory and GLP‐1 concentrations in serum, ileum, and hippocampus.

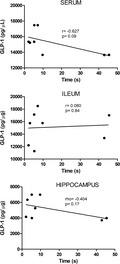

## 1 INTRODUCTION

Cognitive impairment (CI) represents a public health problem. In Mexico, it is estimated that 5–10% of the population aged 60 years or older suffer from some type of CI.^1^

The main mechanisms participating in the induction of CI are those associated with age‐specific pathological changes, such as the production of reactive oxygen species and the decrease in the function of the antioxidant system. These events promote neuronal death^2^ and decrease both spatial memory^3^ and spatial learning.^4^ In addition, numerous studies have strongly correlated the genesis of CI^5^ with a chronic inflammatory state^6^, ^7^ related to dysbiosis which is a condition that causes a significant imbalance of the microbiota‐gut‐brain axis, a bidirectional communication system between the gut microbiota and the brain.^8^

Dysbiosis promotes an increase in the permeability of the intestinal barrier, leading to bacterial translocation and the consequent intestinal inflammation.^9^ This phenomenon alters the blood flow to the blood–brain barrier,^10^ increasing neuroinflammation and promoting neurodegeneration.^11^ Therefore, the restoration of eubiosis through prebiotics and probiotics supplementation could be a therapeutic approach against CI.^12^, ^13^

Prebiotics are selective substrates that promote the growth of beneficial bacteria in the gut.^14^ One example is inulin, which is found mainly in agave^15^ and can attenuate oxidative stress and protein carboxylation.^2^ Additionally, inulin elevates glucagon‐like peptide type 1 (GLP‐1) levels.^16^

On the other hand, probiotics are live microorganisms that improve health when consumed in adequate amounts.^17^ An example is *Enterococcus faecium*, a gram‐positive bacterium naturally present in the gastrointestinal tract. *E. faecium* can confer bacterial antagonism and immune system modulation.^18^

The simultaneous administration of a prebiotic (inulin) and a probiotic (*E. faecium*) –symbiotic‐^14^ could be an interesting strategy for settling dysbiosis and, thereby, improving cognition. In line with this, recent studies have shown that the administration of the symbiotic *E. faecium* + agave inulin improves cognitive function.^19^ However, there is still investigation on the main mechanisms responsible for inducing this beneficial effect. As inulin can increase GLP‐1 levels,^16^ which in turn enhances memory,^20^, ^21^ it is possible to assume that GLP‐1 is the main mechanism through which the symbiotic improves cognition.

The objective of this study was to evaluate the effect of the probiotic *E. faecium*, the prebiotic agave inulin, and the symbiotic (*E. faecium* + inulin) on GLP‐1 levels and its correlation with spatial memory in senile rats.

## 2 METHODOLOGY

### 2.1 Experimental design

This is an analytical and experimental study in which the effect of supplementation with a probiotic (*E. faecium*) and/or a prebiotic (agave inulin) on spatial memory was evaluated in senile rats. For this purpose, 32 rats were randomly assigned into four groups of eight rats each using the GraphPad QuickCalcs program (http://www.graphpad.com/quickcalcs/): Group 1: Control group, to which the vehicle was administered (water at 2 ml/kg); Group 2: *E. faecium* (probiotic) was administered (4 × 10^8^ colony forming units [CFU]/dose);^22^ Group 3: agave inulin was administered (860 mg/kg body weight);^23^ and Group 4: symbiotic group (combination of prebiotic and probiotic at the previously mentioned doses). The sample size for this experiment was calculated using an alpha of 0.05 and a beta of 0.20. This sample size allowed the detection of an effect size of 0.1 or greater.

Supplementation was administered daily for 5 weeks via a 16‐gauge orogastric metal cannula. In the fifth week, a spatial memory evaluation was performed.

The Morris Water Maze (MWM) test was used for analyzing the escape time and the time spent in each of the quadrants. At the end of the experiment, the animals were euthanized with intraperitoneal sodium pentobarbital at a single dose of 1 milliliter (ml). Afterward, the hippocampus, ileum, and serum (by blood centrifugation obtained from intracardiac puncture) were obtained. Subsequently, GLP‐1 levels were quantified using the enzyme‐linked immunosorbent assay (ELISA) technique.

### 2.2 Care of the animals

Thirty‐two adult Sprague–Dawley (15‐month‐old) male rats were used. These rats were kept under a 12‐h light/dark cycle, with a temperature of 20–25°C and humidity between 40% and 70%. The animals were fed with standard Teklad Global Diets® brand pellet feed and ad libitum filter water. The feed was changed every week, the filtered water in the drinking troughs was changed every third day, and the sawdust was changed every third day to maintain hygienic conditions. The animals were visited daily to be supplemented and monitored.

### 2.3 Morris water maze test

This test was used to evaluate spatial memory in rats.^24^ It consists of a circular pool with a hidden platform submerged below the water surface, which must be found by the animal. The location of the platform can only be encoded relative to three visual cues that were positioned equidistant above the water level: thereby using spatial memory. For this study, rats were placed at different starting positions in a circular pool (diameter of 120 cm) filled with water (21–22°C). Rats were trained to find a platform (diameter of 10 cm), which was submerged 2 centimeters below the water surface and located in the southwestern (SW) quadrant of the pool. Animals unable to perform the test were eliminated. The MWM consists of two phases; the first is the acquisition or training phase, in which each rat performed four acquisition trials (maximal swimming time 60 s; 20 s on the platform; inter‐trial interval 20 s) per day for five consecutive days (starting positions varied each day).^25^ All trials were recorded, and latency time, defined as the delay in finding the platform, was used as a measure for spatial learning. During the trial phase, the platform was removed, and the rats were allowed to swim freely for 60 s to analyze the percentage of time spent on the target quadrant (where the platform stood) against the percentage of time spent on the non‐target quadrants. Trials were recorded and analyzed using a computerized system (Smart v3.0.02 Panlab Harvard Apparatus) which calculates the latency time to reach the hidden platform based on the time‐tagged *XY*‐coordinates of the rat.

### 2.4 GLP‐1 determination

The concentration of GLP‐1 was determined by obtaining biological material from three sites: (1) Serum. After administration of pentobarbital in a single dose of 1 ml, an intracardiac puncture (with 1 ml heparinized syringe and insulin needle) was performed in the left paramedian line of the anterior chest wall between the third and fourth rib to obtain 1 ml of blood. Blood samples were stored in 2 ml Eppendorf tubes specifically marked for each group and rat number. The tubes were centrifuged at 4000 revolutions per minute (rpm) for 15 min and maintained at 4°C. The serum was stored in similarly labeled Eppendorf tubes and kept in a freezer at −80°C. For serum analysis, the sample was defrosted at room temperature and a 96‐well microplate was used to perform the ELISA test according to the manufacturer: GLP‐1 (MyBioSource®, USA). Subsequently, the plate was covered with parafilm and placed in a shaker (BioRad®) for 10 min at 120 rpm for mixing and homogenization. The analysis was made by spectrophotometry using the Admin® program, with Thermo Multiskan Spectrum Spectrophotometer, at 500 nm. The bar graphs were made using the Prism 5 program (Prism 5.01, GraphPad Software Inc., San Diego, CA, USA). (2) Intestine: The animal's intra‐abdominal cavity was exposed until the intestine became visible. The cecum was located so that the ileum could be sectioned. The sectioned ileum was stored at −80°C until processing. (3) Hippocampus: After euthanizing the rats, they were decapitated and the brain samples quickly removed. The hippocampus was dissected and weighed. The tissue was stored at −80°C until processing.^26^

After 5 weeks of freezing at −80°C, the homogenization of the samples was carried out. The tissues were rinsed in phosphate‐buffered saline (PBS (0.01 mol/L, pH 7.0–7.2)) in a 10:1 PBS‐weight ratio of tissue to remove excess blood before homogenization. Subsequently, the tissues were chopped into small pieces and homogenized into 5–10 ml PBS with a crystal‐in‐ice homogenizer. The resulting suspension was either sonicated with an ultrasonic cell breaker or subjected to two ice‐thaw cycles to further break down the cell membranes. Afterward, the homogenized samples were centrifuged for 30 min at 14,000 rpm. The supernatants were used for analysis.^27^

The samples were run in duplicate in a spectrophotometer, following the specifications of the ELISA kit: GLP‐1 (MyBioSource®, USA), to measure optical density with a spectrophotometer at a wavelength of 450 nm within 30 min of adding the last solution.^27^

### 2.5 Ethical considerations

The animals were supplied by the animal breeding center, Anahuac University and were handled according to the National Institutes of Health (NIH) guidelines for handling laboratory animals. All procedures were performed in accordance with the NIH Guidelines for the care and use of laboratory animals, and the Mexican Official Standard on technical specifications for the production, care, and use of laboratory animals. All experiments were designed and reported according to the guidelines of “The ARRIVE guidelines” (Animal Research: Reporting of In Vivo Experiments). To perform euthanasia, the animals were previously anesthetized by intramuscular injection with pentobarbital.

The project was evaluated and approved by the CICSA‐Research Committee with the number 201707.

### 2.6 Statistical analysis

The statistical analysis was performed with the Prism 5 program (Prism 5.01, GraphPad Software Inc., San Diego, CA, USA). Data are expressed as mean ± standard error. A descriptive analysis of the four experimental groups was performed. The distribution of the data under the curve was analyzed for normality using the Shapiro–Wilk test. The MWM test was analyzed by two‐way ANOVA. To calculate the size of the effect on spatial memory evaluations, we used Rosenthal's r, an effect size test for data with non‐normal distribution. This test can be used alongside Mann–Whitney *U*‐test results. Results of GLP‐1 concentrations were analyzed with one‐way ANOVA followed by the Tukey test. Correlation analysis between spatial memory and GLP‐1 concentrations in serum and ileum was performed using Pearson's correlation coefficient, for parametric samples. For GLP‐1 concentrations in the hippocampus, Spearman's correlation was used, for non‐parametric samples. Statistical significance was established when *p* < 0.05.

## 3 RESULTS

### 3.1 Spatial memory

**FIGURE 1:**
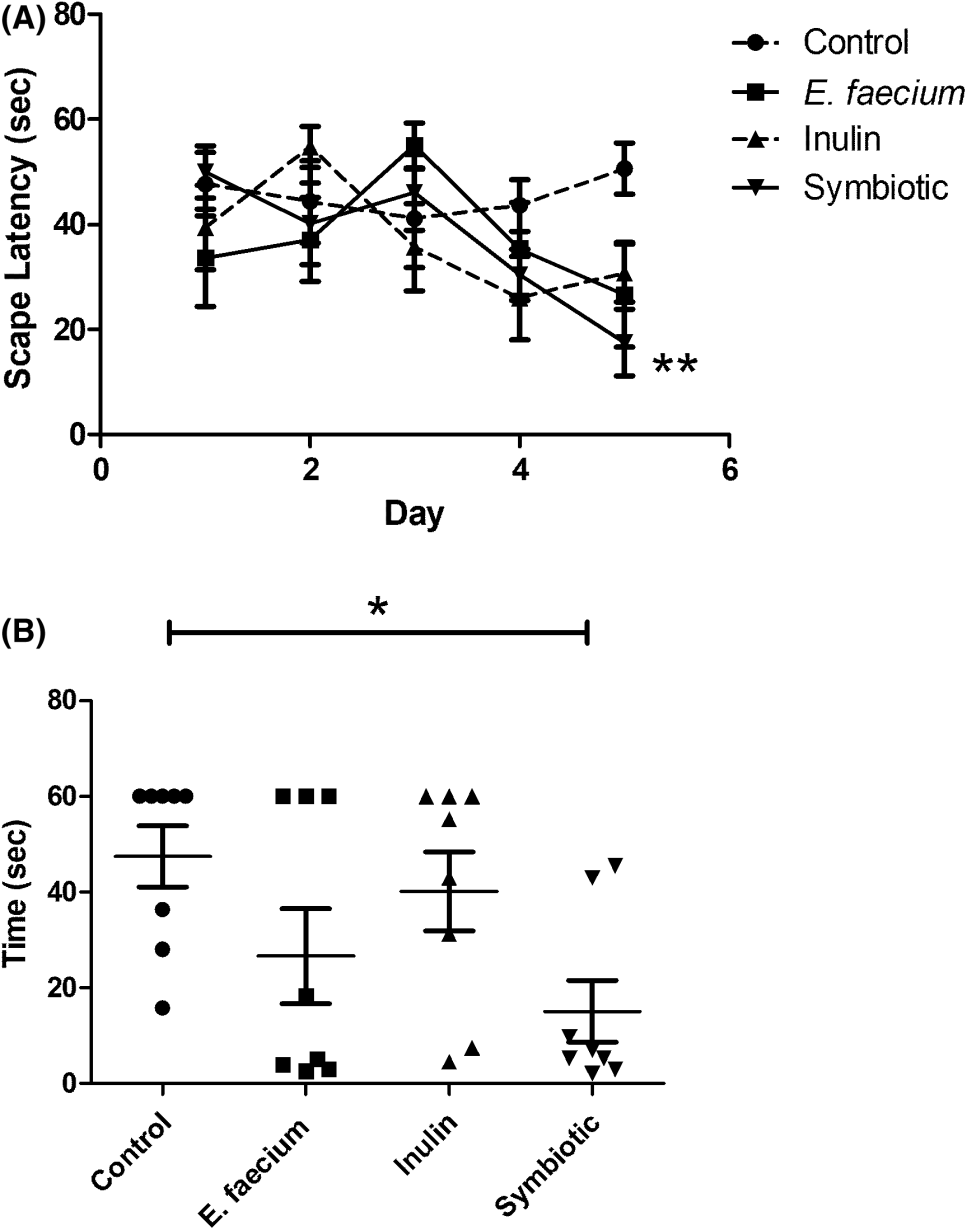
Results from the Morris Water Maze (MWM) test. (A) Escape latency time from the starting point in the northern quadrant and the platform in the southwestern quadrant shows a decrease in time in all supplemented groups. Two‐way ANOVA for repeated measures followed by post hoc Bonferroni (*F*_11,144_ = 1.94, **p = 0.01). (B) On the fifth day of the acquisition phase, the symbiotic group shows a significantly shorter escape time compared to the control group. Mean ± SE, *n* = 8, Mann–Whitney *U* test, *p < 0.05

The analysis of spatial memory revealed a significant decrease in escape latency time in all supplemented groups, especially in those animals treated with the symbiotic. The symbiotic group presented a reduction in time compared to the control group. The interaction between therapy and time was statistically significant (*F*_11,144_ = 1.94, *p* = 0.01; Two‐way ANOVA with Bonferroni post‐hoc test; Figure 1A). On the last day of the acquisition phase, a difference of 32 s was shown between the two groups for escape latency time (control: 47.52 s ± 6.39 [mean ± SE]; symbiotic: 15.13 s ± 6.40; p < 0.05; Mann–Whitney *U* test; Figure 1B). With the aim of strengthening these results, Rosenthal's r was calculated. In this case, a large size effect was obtained in the spatial memory of the symbiotic group (*r* = 0.67).

**FIGURE 2:**
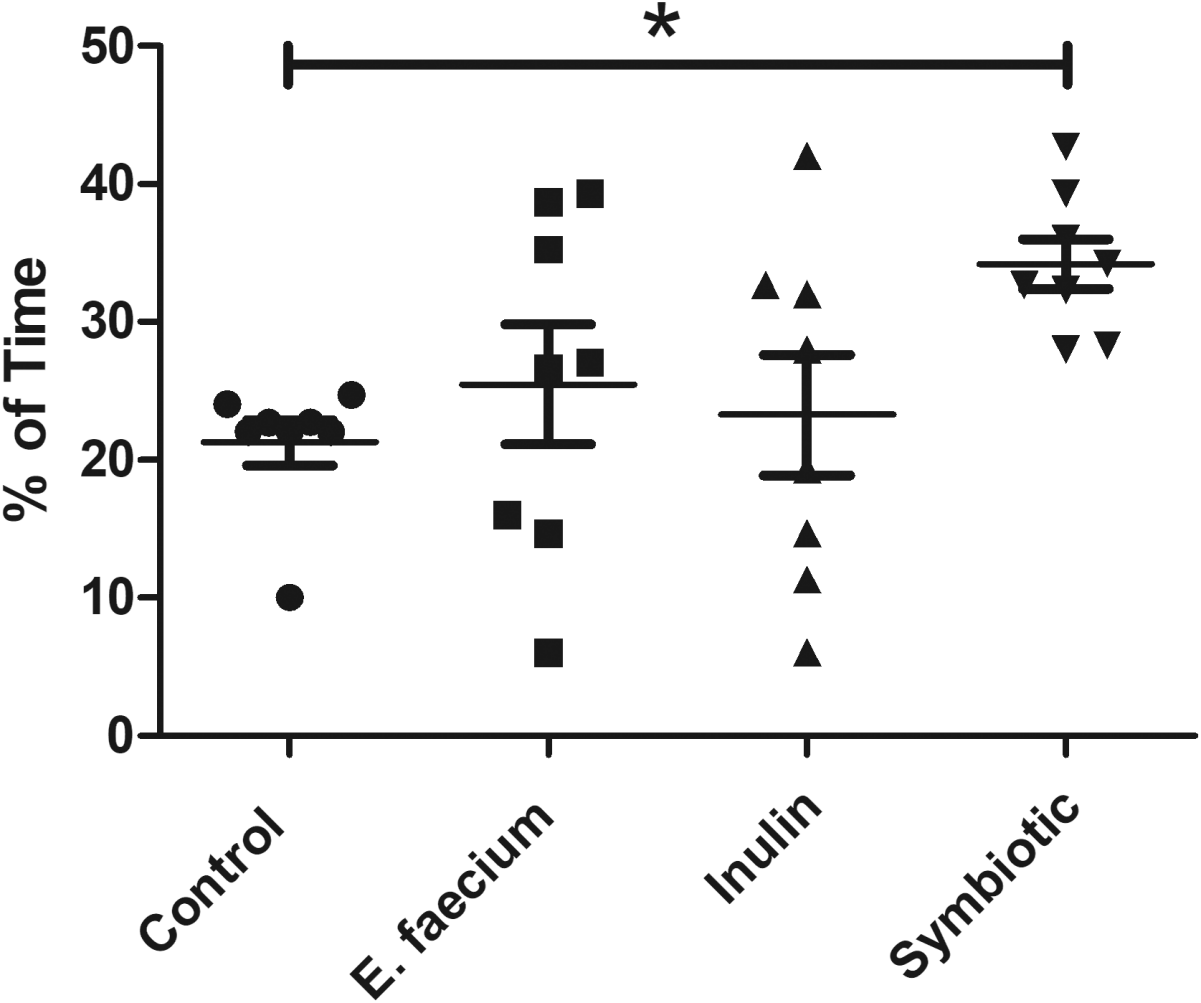
Percentage of time spent in the target quadrant. Time spent in the target quadrant was significantly longer in the symbiotic group. Mean ± SE, *n* = 8. *p < 0.01; one‐way ANOVA with Tukey post‐hoc test

On the sixth day of testing, memory retention was analyzed by removing the platform from the MWM. The time spent in the target quadrant was evaluated. When comparing the control group (21.26 s) with the symbiotic group, the latter had a significantly longer time spent in the target quadrant (34.19 s) (*p* < 0.01; one‐way ANOVA with Tukey post‐hoc test; Figure 2).

### 3.2 GLP‐1 concentration

**FIGURE 3:**
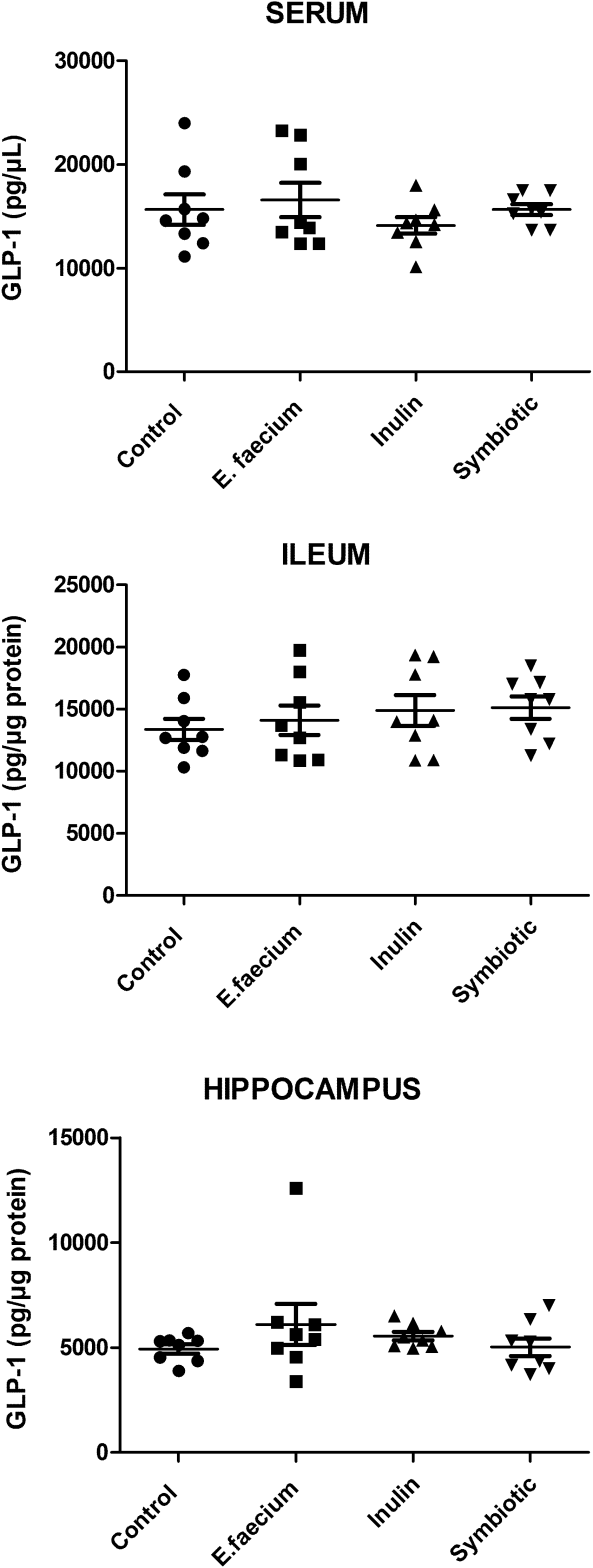
GLP‐1 levels in studied rats. There was no significant difference between the groups. Mean ± SE, *n* = 8, *p* > 0.05; one‐way ANOVA with Tukey Post‐hoc test

Glucagon‐like peptide type 1 levels in serum, ileum, and hippocampus were very similar between the studied groups. There was no significant difference when comparing the concentrations observed in serum (control: 15,807 ± 2002; *E. faecium*: 17,377 ± 2142; inulin: 14,277 ± 1057; symbiotic: 15,233 ± 557; mean ± SE; *p* > 0.05; one‐way ANOVA with Tukey post‐hoc test), ileum (control: 13,763 ± 1109; *E. faecium*: 14,874 ± 1456; inulin: 14,895 ± 1230; symbiotic: 15,126 ± 909; mean ± SE; *p* > 0.05; one‐way ANOVA with Tukey post‐hoc test), and hippocampus (control: 4974 ± 267; *E. faecium*: 5214 ± 506; inulin: 5541 ± 265; symbiotic: 5178 ± 499; mean ± SE; *p* > 0.05; one‐way ANOVA with Tukey Post‐hoc test; Figure 3).

**FIGURE 4:**
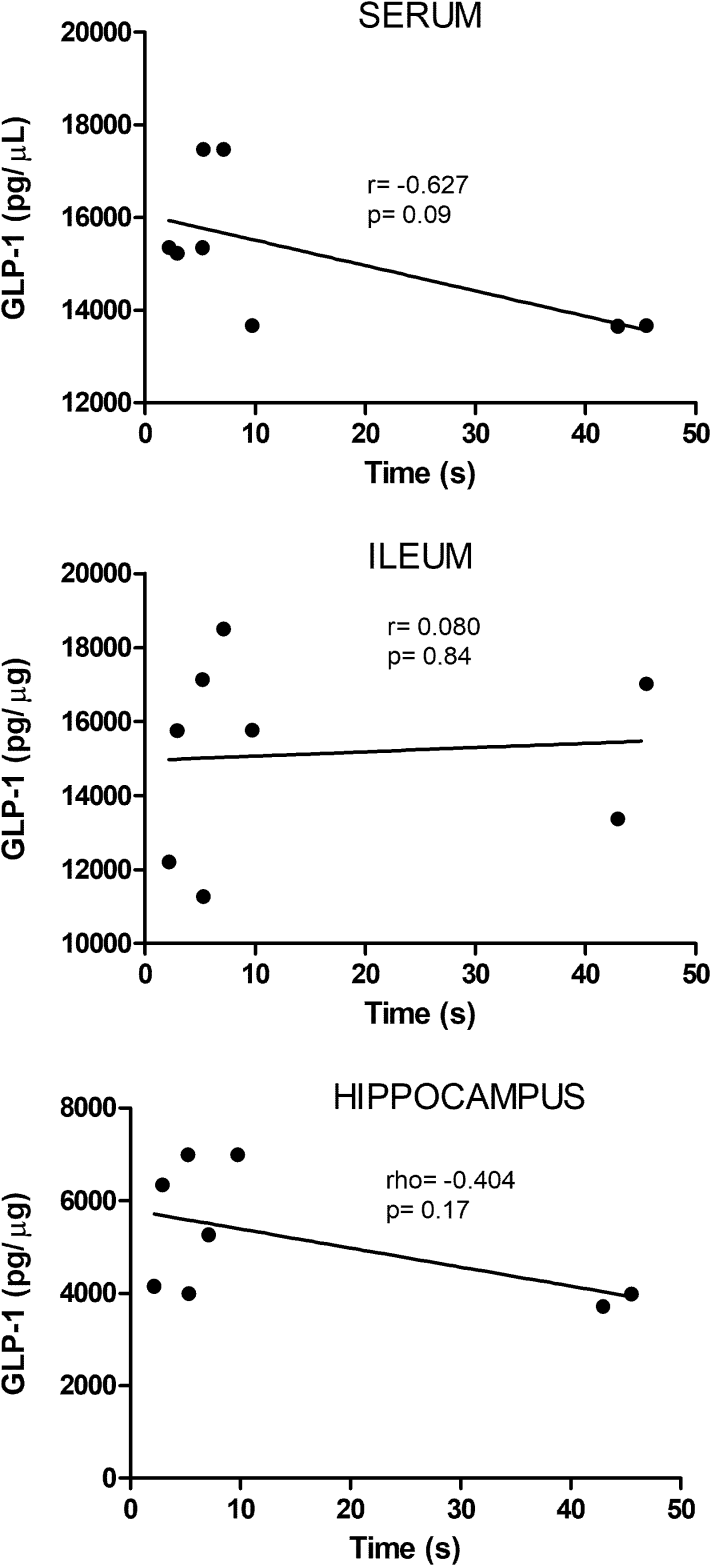
Correlation of spatial memory and GLP‐1 levels in symbiotic‐treated rats. No significant correlation was observed in any case (*p* > 0.05). Correlation analysis between spatial memory and GLP‐1 concentrations in serum and ileum was performed using Pearson's correlation coefficient. For GLP‐1 concentrations in the hippocampus, Spearman's correlation was used

As GLP‐1 has been associated with memory regulation, we decided to correlate the concentrations of this incretin with spatial memory in senile rats.^28^ Therefore, the relationship between these variables was evaluated. Figure 4 shows that GLP‐1 levels in serum (*r* = −0.627), ileum (*r* = 0.080), and hippocampus (rho = −0.404) did not correlate with spatial memory.

## 4 DISCUSSION

Prebiotic and probiotic supplementation has been demonstrated to improve cognitive function. Recent studies have shown that the simultaneous administration of *E. faecium* and agave inulin (symbiotic) promotes better spatial memory.^19^ Although the results of that study were promising and provided a way to explain a possible mechanism of action of this symbiotic, some questions remained about the involvement of other potential elements in improving memory. GLP‐1 could also be related to memory improvement. Upregulation of this incretin in the hippocampus improves cognitive function, promotes neurogenesis, and decreases inflammation and apoptosis.^29^, ^30^ Since the synthesis of GLP‐1 can be stimulated by inulin,^16^ in this study we evaluated the relationship of this incretin with the improvement in memory after the administration of this therapeutic approach.

As shown in the present study, treatment with the symbiotic induced a significant improvement in spatial memory. Nevertheless, this therapeutic approach was not capable of increasing GLP‐1 levels. Furthermore, we found no significant correlation between memory performance and GLP‐1 levels. These findings suggest that, instead of GLP‐1, there could be other mechanisms that participate in the improvement of memory induced by the symbiotic.

Previous studies have shown that memory improvement in animals treated with the same symbiotic we used and at the same dose, was associated with a significant increase in butyrate levels, a short‐chain fatty acid (SCFA) produced by *E. faecium* and which has anti‐inflammatory properties.^19^ Given that neuroinflammation is one of the main factors that induce CI, butyrate production could be reducing inflammation, at least in part, and thus improving cognitive function. On the other hand, inulin could also be modulating inflammation, either by inducing the anti‐inflammatory cytokine interleukin‐10 (IL‐10) or by inhibiting the nuclear factor kappa B transcription factor.^31^ Therefore, the modulation of neuroinflammation could be one of the possible mechanisms that promote memory improvement after the administration of this symbiotic.

Additionally, it has also been reported a significant increase in brain‐derived neurotrophic factor (BDNF) concentration after supplementation with *E. faecium* + inulin.^19^ This molecule could also play an important role in inducing spatial memory improvement. BDNF is a critical molecule for memory consolidation and memory storage.^32^ BDNF is involved in the induction and maintenance of hippocampal long‐term potentiation, which is essential for the encoding and storage of long‐term spatial memory.^19^ Therefore, another possible mechanism of action of symbiotic supplementation could be through the increase in BDNF levels. In this way, anti‐inflammation and BDNF production, independently, could be improving the neurological performance observed in rats supplemented with the symbiotic.

Finally, yet importantly, the lack of response of GLP‐1 should be further studied. According to our results, it could be assumed that the administered dose of the symbiotic could be the cause of the failed response on GLP‐1 levels. Indeed, the upregulation of GLP‐1 by inulin has been reported to be a dose‐dependent effect.^33^ Studies in which inulin increased GLP‐1 levels administered higher amounts of this prebiotic than those used in our study.^34^ The latter could be the main reason why, in this study, inulin did not increase GLP‐1 levels.

The present study confirms that GLP‐1, a possible promoter of memory enhancement, does not participate in the improvement of spatial memory observed in rats supplemented with the symbiotic formed by *E. faecium* and inulin.

## 5 CONCLUSIONS

The symbiotic consisting of *E. faecium* and inulin induces a significant improvement in spatial memory in middle‐aged rats. This memory improvement did not correlate with GLP‐1 levels. Our study supports the inclusion of other mechanisms of action of symbiotics that lead to memory improvements, such as BDNF production and anti‐inflammatory effects.

## AUTHOR CONTRIBUTIONS

All authors contributed equally to the following three components: (1) concept and design of study or acquisition of data or analysis and interpretation of data; (2) drafting the article or revising it critically for important intellectual content; and (3) final approval of the version to be published.

## FUNDING INFORMATION

This work was supported by funds and infrastructure of Universidad Anáhuac México.

## CONFLICT OF INTEREST

The authors declare not to have any conflict of interest.

## Data Availability

Research materials related to this manuscript will be provided upon request to the authors.
